# Supplementary material for: Identifying SARS-CoV-2 antiviral compounds by screening for small molecule inhibitors of Nsp14 RNA cap methyltransferase
Source: Biochem J. 2021 Jul 2;478(13):2481–97. doi: 10.1042/BCJ20210219 (PMC8286817; doi:10.1042/BCJ20210219)
Supplement: Supplementary Figures S1-S4 [file BCJ-478-2481-s1.pdf]

Supplementary Figures

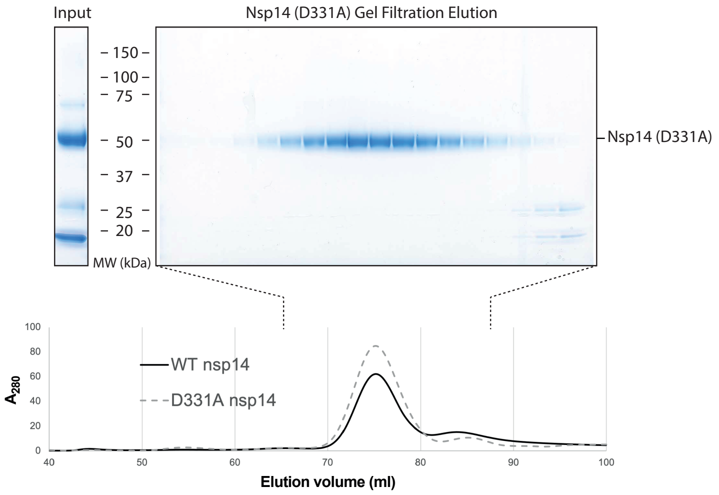

Supplemental Figure 1 (S1)– Purification of nsp14(D331A)

Upper: Gel filtration fractions for nsp14(D331A) across the main elution peak from size-exclusion chromatography (lower). Nsp14 expected size: 55 kDa. Input to gel filtration column is given in the left most panel. Lower: Nsp14(D331A) elution profile superimposed on nsp14(WT) elution profile.

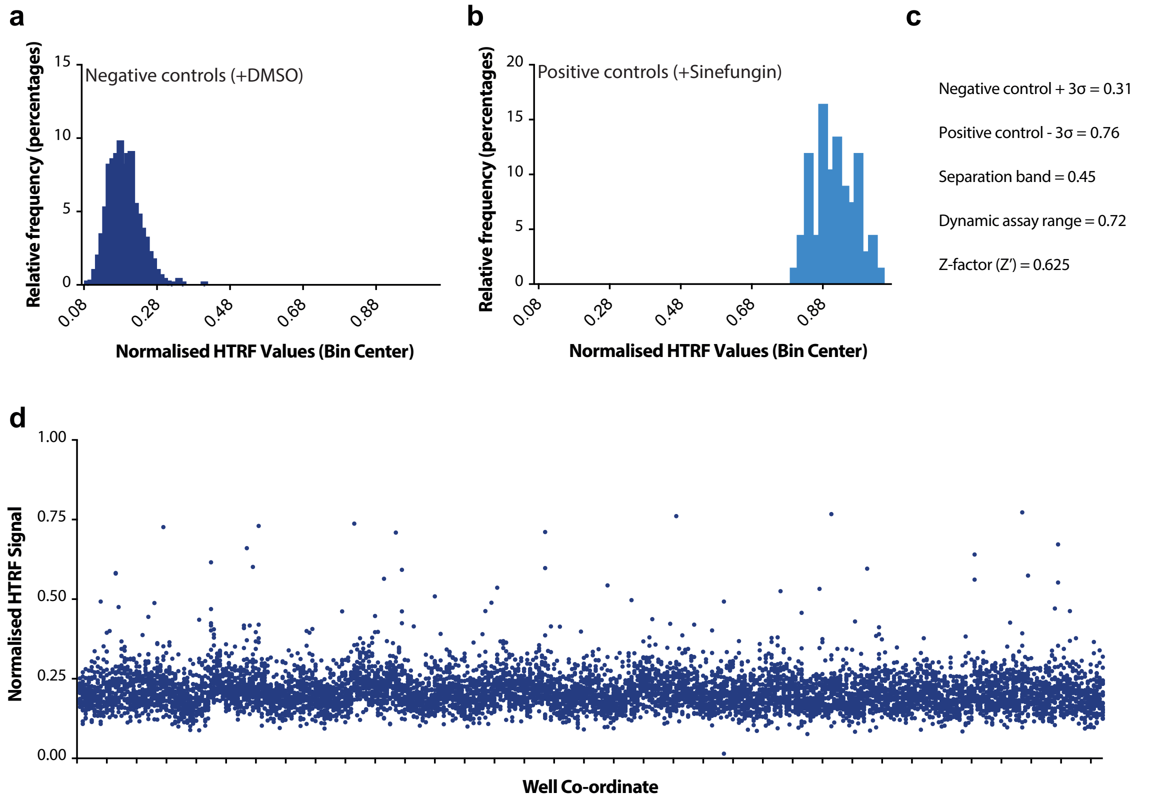

Supplemental Figure 2 (S2)– Screening statistics

a) Normalised HTRF values for negative controls (+DMSO) within screen plates. N = 822.

b) Normalised HTRF values for positive controls (+ Sinefungin) within screen plates. N = 67.

c) Screening statistics as determined from the data in panels **(a)** and **(b)**.

d) Raw normalised HTRF values over all compounds. Compounds are ordered by screening order, from first value read onwards.

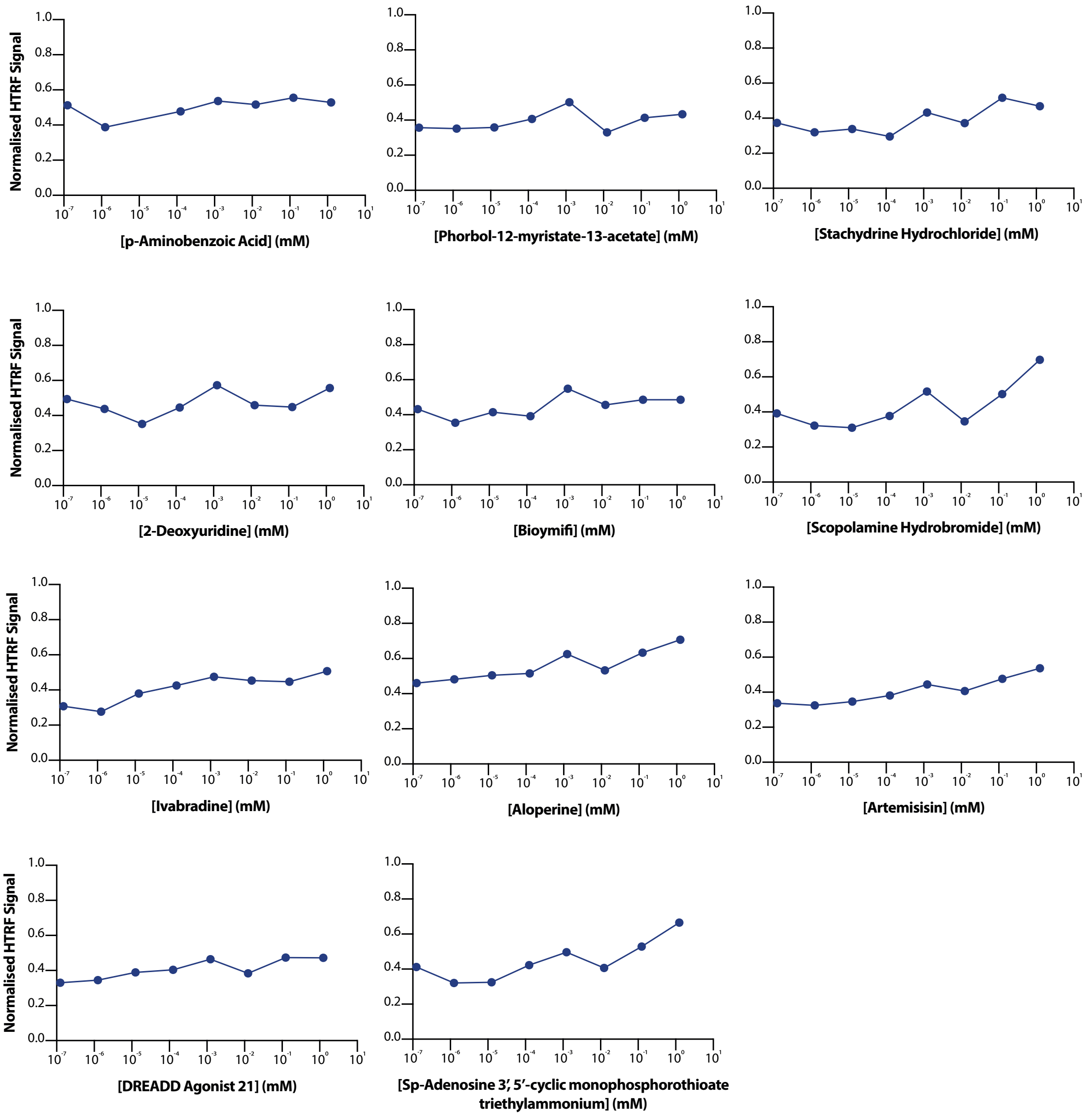

Supplemental Figure 3 (S3)– Validation of other hit compounds

Normalised HTRF values for other screen hit compounds in validation. All reactions were run with 10 nM nsp14, 1 µM SAM, and 0.11 mM GpppA cap analogue for 20 minutes at room temperature before quenching with NaCl (see methods)

PF-03882845	Inauzhin	Lomeguatrib	Trifluperidol
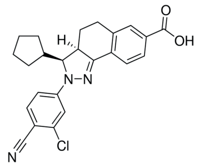	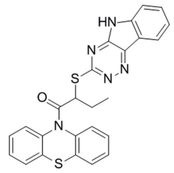	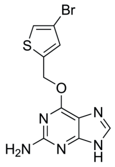	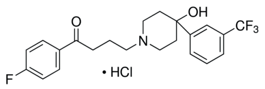

Supplemental Figure 4 (S4)– Chemical structures of nsp14 inhibitors

Chemical structures of nsp14 inhibitors validated in HTRF assays and in cellular infection models.

Supplementary Tables

Supplemental Table 1 (Table S1)– Hit compounds from screening

List of 63 compounds that were considered “hits” from primary screening, and their associated Z-scores.
